# HPV16 E6 gene variations in invasive cervical squamous cell carcinoma and cancer in situ from Russian patients

**DOI:** 10.1054/bjoc.2000.1619

**Published:** 2001-03

**Authors:** X Hu, T Pang, Z Guo, N Mazurenko, F Kisseljov, J Pontén, M Nistér

**Affiliations:** Department of Genetics and Pathology, Rudbeck Laboratory, Uppsala University, SE-751 85 Uppsala, Sweden; Blokhin Cancer Research Center, Moscow, 115478, Russia

**Keywords:** HPV, sequence variation, cervical cancer, cancer in situ

## Abstract

HPV16 is frequently seen in invasive cervical cancer (ICC) and cervical intraepithelial neoplasia (CIN). Its E6 gene has frequent sequence variations. Although some E6 variants have been reported to have different biochemical or biological properties, they do not show geographical identity. Moreover, the definition of ‘variant’ has been a source of confusion because it has been based on all departures from the ‘prototype’ once isolated randomly from an ICC case. We amplified the HPV16 E6 gene by PCR from fresh-frozen tissue of 104 cases of ICC and CIN from Russian patients and sequenced it in positive cases. We found that 32 of 55 (58.2%) ICC cases and 18 of 49 (36.7%) CIN cases were HPV 16-positive and we could identify 3 groups of E6 variants: group A was characterized by G at nt 350 where group B had T, and group M was a heterogeneous mixture of unique E6 variants; no significant difference existed in the distribution of the different groups between ICC and CIN; the clinically malignant (as defined by FIGO stage) order between the groups was M > A > B in ICC; in the cases with a single HPV16 E6 sequence, coexisting ICC, CIN and normal epithelium in the same patient shared the E6 variant; and 4 cases of ICC had double/multiple E6 variants. The results did not show any importance of E6 variants for ICC progression in Russian women. The results also indicated that the original HPV16 variant persisted during ICC progression, and that at a low frequency, double infections and/or mutation of variants might occur. © 2001 Cancer Research Campaign http://www.bjcancer.com


*British Journal of Cancer* (2001) **84**(6), 791–795
© 2001 Cancer Research Campaign
doi: 10.1054/ bjoc.2000.1619, available online at http://www.idealibrary.com on IDEAL®

http://www.bjcancer.com

### HPV16 E6 gene variations in invasive cervical squamous cell carcinoma and cancer in situ from Russian patients

**X Hu[1], T Pang[1], Z Guo[1], N Mazurenko[2], F Kisseljov[2], J Pontén[1] and M Nistér[1]**

[1] Department of Genetics and Pathology, Rudbeck Laboratory, Uppsala University, SE-751 85 Uppsala, Sweden; [2] Blokhin Cancer Research Center, 115478, Moscow, Russia

**Summary** HPV16 is frequently seen in invasive cervical cancer (ICC) and cervical intraepithelial neoplasia (CIN). Its E6 gene has frequent sequence variations. Although some E6 variants have been reported to have different biochemical or biological properties, they do not show geographical identity. Moreover, the definition of 'variant' has been a source of confusion because it has been based on all departures from the 'prototype' once isolated randomly from an ICC case. We amplified the HPV16 E6 gene by PCR from fresh-frozen tissue of 104 cases of ICC and CIN from Russian patients and sequenced it in positive cases. We found that 32 of 55 (58.2%) ICC cases and 18 of 49 (36.7%) CIN cases were HPV 16-positive and we could identify 3 groups of E6 variants: group A was characterized by G at nt 350 where group B had T, and group M was a heterogeneous mixture of unique E6 variants; no significant difference existed in the distribution of the different groups between ICC and CIN; the clinically malignant (as defined by FIGO stage) order between the groups was M > A > B in ICC; in the cases with a single HPV16 E6 sequence, coexisting ICC, CIN and normal epithelium in the same patient shared the E6 variant; and 4 cases of ICC had double/multiple E6 variants. The results did not show any importance of E6 variants for ICC progression in Russian women. The results also indicated that the original HPV16 variant persisted during ICC progression, and that at a low frequency, double infections and/or mutation of variants might occur. © 2001 Cancer Research Campaign http://www. bjcancer.com

**Keywords:** HPV; sequence variation; cervical cancer; cancer in situ

HPV (Human Papilloma Virus) 16 is the most frequent HPV type found in invasive cervical carcinoma (ICC) in Russian women (Samoylova, 1995; van Muyden, 1999). Although infection with HPV16 and some other high/intermediate-risk types of HPV such as HPV18, 31, 33 etc. is considered a main risk factor for ICC and cervical intraepithelial neoplasia (CIN), only a minority of women with infection by these types of HPV develop any disease (Munoz et al, 1992; Eluf et al, 1994; Bosch et al, 1995; Matsukura and Sugase, 1995). Specific HPV16 variations located in E6, E7, E2, E5, L1, L2 or the long control region (LCR) have been associated with viral persistence and development of high-grade cervical lesions (Xi et al, 1995; Yamada et al, 1995; Londesborough et al, 1996; Wheeler et al, 1997). HPV16 E6 and E7 encode oncoproteins able to interact with regulatory proteins such as p53 and pRb; they are regarded as the major if not the only genes responsible for neoplastic transformation (Dyson et al, 1989; Werness et al, 1990; Phelps et al, 1992; von Knebel Doeberitz et al, 1994).

Sequence variations are frequent particularly in HPV16 E6 (Alvarez-Salas et al, 1995; Xi et al, 1995; Yamada et al, 1995–1997; Londesborough et al, 1996; Xi et al, 1997; Zehbe et al, 1998). The variants are reported to have different biological and biochemical properties. Londesborough found that only 1 of 16 women infected with the HPV16 prototype developed CIN or ICC; in contrast, 10/12 women infected with HPV16 E6 variants had persistent infection which is associated with development of CIN and ICC (Londesborough et al, 1996). Zehbe concluded that sequence variation in HPV16 E6 predicted risk of progression from CIN III, because 15/16 cases of ICC contained variant E6 in contrast to CIN III where only 11/25 had variant E6 (Zehbe et al, 1998). Alvarez-Salas showed that variants of HPV 16 E6 correlated positively with clinical aggressiveness (Alvarez-Salas et al, 1995). Stöppler displayed that variants of HPV 16 E6 protein differed in the abilities to suppress keratinocyte differentiation and to induce P53 degradation in vitro (Stoppler et al, 1996). However, the rate, the type and the biological and clinical significance of the variations in HPV 16 E6 have not been geographically uniform, which might be explained by the geographically different distribution of the HLA polymorphisms (Ellis et al, 1995). The HPV 16 E6 variations in cervical samples from Russian women has not been studied previously and in this work we have examined HPV 16 E6 in ICC and CIN from Russian pattients.

The definition of 'variant' has been a source of confusion in previous studies, because it has been based on all departures from the original 'prototype' once isolated randomly from a case of ICC. Among the 50 HPV16-positive cases out of 104 Russian patients, we show that there were two major forms of E6, one of which was the international prototype, as reported by other studies (Yamada et al, 1997; Zehbe et al, 1998). They differ from each other only at nt 350 with either a guanine (29/50 cases) or a thymidine ('prototype', 9/50 cases) residue. A third heterogeneous variant group (12/50 cases) with apparently genuine mutations on the background of the two major forms was defined. Application of this scheme failed to substantiate that different configurations of E6 determine progression from CIN to ICC among Russian patients.

*Received 5 April 2000*
*Revised 7 November 2000*
*Accepted 8 November 2000*

*Correspondence to:* X Hu

### MATERIALS AND METHODS

### Patients

We collected 104 cases, including 55 cases of primary invasive squamous cell carcinoma and 49 cases of CIN, from patients undergoing radical hysterectomy at the Blokhin Cancer Research Center, Moscow, Russia in the period from 1994 to 1998. Among the collected cases, the HPV16-positive cervical specimens as determined by PCR analysis were selected to perform the HPV16 E6 sequence variation test, where 32 out of 55 (58.2%) ICC cases and 18 out of 49 (36.7%) CIN cases were HPV16 positive. The age range was 30–80 (mean 43.8) for the HPV16-infected patients with ICC and 31–43 (mean 37.2) for those with CIN. The FIGO (the standard of the International Federation of Gynecology and Obstetrics subdivides the cervical carcinoma cases into stages I to IV) (Sparen et al, 1995) stage and degree of differentiation (high, moderate or low) were recorded at the Blokhin Cancer Research Center.

Tumour samples were snap-frozen in liquid nitrogen. Part of each sample was transferred on dry-ice to the Department of Genetics and Pathology, Uppsala University, Sweden. This project had received official institutional and ethical approval.

### Microdissection and DNA preparation

Sections (6 μm) were prepared from the fresh tissue and stained with Mayer's haematoxylin. CIN and invasive cancer nests were microdissected (Hedrum et al, 1994). CIN presented simultaneously with invasive cancer in 7 surgical specimens, so multiple microdissections were then performed from CIN, the invasive cancer and normal squamous epithelium. 7 other cases of invasive cancer were also multiply microdissected. All lesions were sharply demarcated from stroma or adjacent normal epithelium. Admixture by normal cells was insignificant as judged by examination under microscope. The blade of the scalpel was changed after each microdissection. The dissected pieces were transferred to Eppendorf tubes containing 30 μl 1 × PCR buffer II (PE, Roche Molecular System, NJ). Each sample contained 500–1000 cells. Digestion by proteinase K (500 μg $ml^{-1}$) at 55˚C for 4 hours was interrupted by incubation at 95˚C for 10 min. Quality of the prepared DNA was checked by PCR amplification of microsatellite markers.

### Polymerase chain reaction

PCR primers for HPV16 E6 were 5′-CGTAACCGAAATCG-GTTGAAC-3′ and 5′-GCTCATAACAGTAGAGATC-3′ (Yamada et al, 1995). We performed PCR on a RoboCycler Gradient 96 (STRATAGENE) in 50 μl volume (1 × PCR buffer II, 2.5 mM $MgCl_2$, 200 μM of each deoxynucleotide, 0.5 U Taq DNA polymerase (PE, Roche Molecular System, NJ), 0.5 μM of each sense and anti-sense primer, and 5 μl DNA solution) with a 35 cycle protocol: 1 min denaturation at 95˚C, annealing at 55˚C and extension at 72˚C, with 5 min initial denaturation at 95˚C and 7 min final elongation at 72˚C.

To avoid contamination, we prepared PCR master mix in an isolated room under a hood where UV light was used to destroy any potential contaminating DNA or PCR product at the working area before and after this manipulation and then added template DNA under similar working conditions in a separate room.

### Sequence analysis

PCR amplicons were electrophoretically separated on 1.5% agarose gel and stained with ethidium bromide. Desired bands were cut out with subsequent purification on GenElute Minus EtBr Spin Columns (SUPELCO, Bellefonte, PA). The purified PCR products were quantified and then applied to enzymatic extension reactions for DNA sequencing using the Cycle Sequencing Ready Reaction Kit (Big-Dye terminator reagent (PE Applied Biosystems) containing dye-labelled terminators) in GeneAmp PCR Systems 9600 (PE, Norwalk, CT). The same forward and reverse primers as for the PCR amplification of E6 were used separately in cycle sequencing. The extension products were purified by ethanol/-sodium acetate precipitation, then electrophoresed on an ABI Prism 377 sequencer. The sequence and variations were analysed and determined by the FacturaTM and Sequence Navigator version 2.0 (PE Applied Biosystems).

The test was repeated once for each sample starting from DNA-PCR amplification with the same result.

### Statistical analysis

The Chi-square test was used to assess the relation of the prevalence of different variant groups between ICC and CIN. The rank sum test was used to judge the relation of the variants with the FIGO stages.

### RESULTS

### Prevalence of HPV 16 E6 variations

32 cases of ICC and 18 cases of CIN were analysed for HPV16 E6 sequence variation (Figure 1).

Any E6 sequence isolated was here defined as variant. All CIN and 28 out of 32 ICC cases had a single E6 variant; the remaining 4 cases of ICC carried double/multiple E6 variants. The E6 variants were classified as three groups, A, B and M. A and B were identical except for the polymorphic nt 350, which was either guanine (group A) or thymidine (group B). Group M was a mixture of variants with sequence departures from group A and/or B at other sites than nt 350. The 4 cases with double/multiple E6 isolates were assigned to group M.

Group A dominated both in cases of ICC (19/32; 59.4%) and cases of CIN (10/18; 55.5%). Group B, which corresponded to the international 'prototype', occurred in 5/32 (15.6%) cases of ICC and 4/18 (22.2%) cases of CIN. The third group (M) was represented by 8/32 (25%) cases of ICC and 4/18 (22.3%) cases of CIN. There was no statistically significant difference in the distribution of groups A, B and M between CIN and ICC.

Many studies categorize HPV16 E6 sequences into only two groups, 'prototype' and 'variants' group. When these previous categories were applied to our data, the 'variants' group accounted for 27/32 (84.4%) cases of ICC and 14/18 (77.7%) cases of CIN, respectively. No significant difference in the distribution of the 'prototype' group and 'variants' group was found between CIN and ICC.

### Relation of HPV 16 E6 variants to the FIGO stages of ICC

Among 32 cases of ICC, 4 cases in group A and one case in group M were unknown for FIGO stage. The relation of HPV16 E6

 © 2001 Cancer Research Campaign

| | Open reading frame | | | | | | | | | | | | | Predicted amino acid | Number of cases CIN | ICC | |
|---|---|---|---|---|---|---|---|---|---|---|---|---|---|---|---|---|---|
| @ | 1 | 1 | 1 | 2 | 2 | 2 | 2 | 3 | 3 | 3 | 3 | 4 | 5 | | | | |
| | 0 | 4 | 8 | 1 | 7 | 8 | 8 | 1 | 3 | 5 | 7 | 5 | 3 | | | | |
| | 9 | 5 | 8 | 5 | 6 | 6 | 9 | 0 | 5 | 0 | 4 | 2 | 2 | | | | |
| # | T | G | G | C | A | T | A | T | C | T | C | A | A | | | | |
| **A** | – | – | – | – | – | – | – | – | – | G | – | – | – | L83V | 10 | 19 | |
| **B** | – | – | – | – | – | – | – | – | – | – | – | – | – | – | 4 | 5 | |
| **M** | – | – | A | – | – | – | – | – | – | G | – | – | – | E29K/L83V | 1 | 0 | |
| | – | – | – | T | – | – | – | – | – | G | – | – | – | L38L/L83V | 1 | 0 | |
| | – | – | – | – | T | – | – | – | – | – | – | – | – | N58I | 1 | 0 | |
| | – | – | – | – | – | – | – | – | – | G | – | T | – | L83V/R117G | 1 | 0 | |
| | C | – | – | – | – | – | – | – | – | G | – | – | – | F2F/L83V | 0 | 1 | |
| | – | T | – | – | – | A | G | – | T | G | – | – | – | Q14H/A61A/V62V/H78Y/L83V | 0 | 2 | |
| { | – | – | – | – | – | – | – | – | – | G | – | – | – | L83V | | | |
| { | – | – | – | – | – | – | – | – | – | – | – | – | – | – | 0 | 1 | (M13) |
| { | – | – | – | – | – | – | – | G | – | – | – | – | – | F69L | | | |
| { | – | – | – | – | – | – | – | – | – | G | – | – | – | L83V | | | |
| { | – | – | – | – | – | – | – | – | – | – | – | – | – | – | 0 | 2 | (M4, M12) |
| { | – | – | – | – | – | – | – | – | – | G | – | – | – | L83V | | | |
| { | – | – | – | – | – | – | – | – | – | – | – | – | – | – | 0 | 1 | (M23) |
| { | – | – | – | – | – | – | – | – | – | – | T | – | – | Q91Stop codon | | | |
| { | – | – | – | – | – | – | – | – | – | G | – | – | G | L83V/S143S | 0 | 1 | |
| | | | | | | | | | | | | | | | 18 | 32 | |

**Figure 1** Sequence variations of HPV 16 E6 in CIN and ICC. @: nucleotide positions are indicated vertically, e.g., 109, 111, and so forth. #: reference nucleotides. –, presents the reference nucleotide at this position. The position of amino acid is stated numerically. The letter preceding this number refers to the reference amino acid, and the letter after it refers to the amino acid predicted from the nucleotide sequence found. A, B and M, are the given names of the variant groups. The square brackets to the left group together the multiple variants found in M13, M4, M12 and M23, respectively

**Table 1** Relation of HPV16 E6 variant groups with FIGO stage

| FIGO stage | A | B | M |
|---|---|---|---|
| Ia | 2 | 2 | 0 |
| Ib | 3 | 2 | 0 |
| IIa | 3 | 1 | 1 |
| IIb | 1 | 0 | 1 |
| III | 5 | 0 | 3 |
| IV | 1 | 0 | 2 |
| Total | 15 | 5 | 7 |

Rank sum test (two sides), $P < 0.05$ between A and B; $P < 0.05$ between A and M; and $P < 0.005$ between B and M.

variants to the FIGO stage of ICC is shown in Table 1. Group B was mainly associated with cancers detected in early clinical stages. Group A, the most common one, was represented in early as well as advanced stages. The M group was not seen in FIGO stage I. Differences between the groups were statistically significant indicating that the order of clinical malignancy between the groups would be M > A > B.

### HPV 16 E6 variants at nt 350 in synchronous lesions or multiple samples of ICC

Since nt 350 was the most common polymorphic site, this section would mainly focus on description of nucleotide variations at this site in 14 out of 32 cases of ICC with synchronous lesions or multiple samples available (Table 2). Among these cases, 10 contained a single HPV16 E6 isolate. The E6 variant in these cases was concordant from different parts of each ICC case. When CIN was also present (N3, M2, M21), all samples from these precursors had the same variant as in the simultaneously found invasive cancer. Normal squamous epithelium sampled in case M19 and N3 showed the same variant as in the invasive cancers.

4 cases (M4, M12, M13, M23) carried double/multiple E6 variants, M12 was an invasive cancer with G (one sample), T (one sample) or G + T (one sample). In M4, the CIN II lesion had either G (4 samples) or T (one sample); the synchronous invasive cancer had G in 2 but T in one sample; and the variants were identical in the CIN II samples and the samples from the invasive cancer. M13 presented T in 3/3 samples from its CIN II; and in the invasive cancer, one sample showed G and another sample T at nt 350 with an additional missense variation from T to G at nt 310. M23 showed T in the only sample from normal epithelium; of two CIN II samples, one showed G at nt 350, another showed T at nt 350 with an additional change of C to T at nt 374 which created a stop signal; the invasive cancer had either T (2 samples) or G (2 samples).

Double/multiple HPV16 E6 variants detected in a single case might be considered as the result of PCR artifacts or contamination. However, this could be easily ruled out because of the following facts: the specimens used were fresh specimens which are quite safe not to introduce PCR artifacts (Williams et al, 1999); every step of the PCR preparation procedure was manipulated very strictly in order to avoid any potential contamination; the test was repeated with the same result; there were 4 cases with double/multiple variants; more than one sample in M4, M13 and M23 had E6 variants with either G or T at nt 350; out of 23 samples from these 4 cases, 22 showed the variant with a single G or T signal at nt 350, and only one showed G + T which might occur at a time when the microdissected sample is derived from 2 overlapping sets of cells with different HPV variants.

*© 2001 Cancer Research Campaign*

**Table 2** HPV 16 E6 variants at nt 350 in synchronous lesions and multiple microdissected samples of invasive cancer

| Case | Normal | CIN I | CIN II | CIN III | ICC |
|---|---|---|---|---|---|
| M1 | – | – | – | – | G 3/3 |
| M3 | – | – | – | – | G 3/3 |
| M6 | – | – | – | – | G 2/2 |
| M8 | – | – | – | – | G 2/2 |
| M15 | – | – | – | – | G 2/2 |
| M25 | – | – | – | – | G 2/2 |
| M19 | G 2/2 | – | – | – | G 2/2 |
| N3 | G 2/2 | G 1/1 | G 2/2 | G 2/2 | G 4/4 |
| M2 | – | – | – | T 1/1 | T 3/3 |
| M21 | – | – | T 1/1 | – | T 4/4 |
| M12 | – | – | – | – | G 1/3, T 1/3, T+G 1/3 |
| M4 | – | – | G 4/5, T 1/5 | – | G 2/3, T 1/3 |
| M13 | – | – | T 3/3 | – | T 1/2, G 1/2 |
| M23 | T 1/1 | – | T 1/2, G 1/2 | – | T 2/4, G 2/4 |

The fractions indicate number of samples with the indicated nucleotide over total number of samples. See text and Figure 1 for variations at other sites than nt 350. –, no lesions of this type available.

### DISCUSSION

The present HPV16 E6 variations in Russian patients were similar to other results (Londesborough et al, 1996; Yamada et al, 1997; Zehbe et al, 1998). The pattern of nucleotide substitutions leads us to propose 3 categories of sequences in the HPV16 E6 genome. Some previous investigators have used the ‘prototype’ sequence, which corresponds to our group B, as a yardstick and labelled all departure ‘variants’. We found that the nucleotide sites differ principally from each other. Nt 350 behaves as a polymorphic site where roughly two thirds of the isolates have a G and the remainder a T. The other sites register as classical variants with one or occasionally two departures from a predominant configuration. This classification permits a logical division into two homogenous groups (A and B) supplemented by one heterogeneous group M. Failure to have noted this is the major reason for a confused literature based on lumping together all ‘pure non-T’ at nt 350 with all other departures from the sequence of the prototype. When our groups A, B and M are applied, the distribution of the 3 groups in invasive cancer and CIN is identical. This contrasts with claims that E6 variants at nt 350 have a higher prevalence in invasive cancer than in CIN III. Therefore, our findings do not support the conclusion that ‘variants’ of E6 are more likely to cause progression to invasive cancer than ‘prototype’ E6 (Zehbe et al, 1998).

Even if the previous scheme (Zehbe et al, 1998) which classified E6 variants as two groups, ‘prototype’ group and ‘variants’ group, is applied on our cases, no difference in the distribution of the E6 groups can be seen between CIN and ICC in cervical samples from Russian patients.

Sampling bias might affect the judgement of the E6 variant distribution. We collected 104 cases of CIN and ICC from Moscow during the period from 1994 to 1998. Our cases seem to be representative of the selected population because 58.2% of ICC and 36.7% of CIN samples were found to be HPV16 positive, which is similar to the results of other larger studies on Russian cases and world wide (Bosch et al, 1995; Matsukura and Sugase, 1995; van Muyden, 1999).

Circulating HPV16 E6 variants might shift during a specified period of time. Among our cases, the mean age of patients with ICC was 6.6 years higher than that of patients with CIN, which means ICC and CIN might carry different circulating E6 variants from different periods. Unfortunately, we could not exclude this possibility concerning fluctuations in the distribution of HPV16 variants over time within the population. The question if such fluctuations do occur has not been addressed in detail in the literature.

Our results are consistent with many other recent findings that HPV16 E6 ‘variants’ and ‘prototype’ have an equally malignant potential (Bontkes et al, 1998; Luxton et al, 2000). To finally elucidate the E6 variants-associated disease outcomes, longitudinal cohort studies should be conducted. To some extent, the multiple microdissections of the synchronous lesions performed here might mimic longitudinal cohort studies. The use of E6 genomic markers has permitted an insight into persistence of the same variant in different lesions of the same patient. The overall impression is that the same variant will be present in the entire chain leading from normal epithelium via CIN to invasive cancer in the cases with a single E6 sequence variant. These findings are identical to conditions in Swedish women (Hu et al, 1999) and support the relevance of the cross-sectional study.

In 4 cases (M4, M12, M13 and M23) of ICC, mixtures of two or more different E6 genomes are disclosed. Two explanations can be offered: either the patients have been multiply infected or the second and/or third variant is/are derived from the original HPV16 isolate by mutations within the patient herself. In general, the common polymorphic variations at nt 350, T or G, are less likely to have been substituted for each other within a certain patient because the mutation at this site might have occurred long ago, and then either T or G was naturally selected and kept stable to prevalently circulate. Since T and G variants at nt 350 of E6 are so common, it should not be difficult for one patient to pick up both variants in repeated infections. M12 presents two different E6 genomes in the invasive cancer, and M4 shows two E6 variants both in CIN and invasive cancer. These two cases could represent double infections. In contrast, the results of M13 and M23 were much more complicated. It seems to us that the results were compatible with events where the second and/or third variants were derived from the original infection by mutations. M13 seems to have a putative parental E6 variant with T at nt 350 in the invasive cancer, and in this variant a mutation at nt 310 from T to G occurs. M23 seems to be originally infected by an E6 variant with T at nt 350 which presents in the normal epithelium, in the

© 2001 Cancer Research Campaign

CIN II lesion and invasive cancer, and then occurs a mutation at nt 374 from C to T in CIN II. The reason for this is unclear. We have not been able to ascertain whether the E6 DNA is integrated or present in episomal form. In the former situation, the E6 genome would be subject to the same genomic instability as the cellular genome of the cancer cell (Mazurenko et al, 1999). Since the E6 variants fail to show importance in the progression of ICC, the positive association of the M group with clinical malignancy (Table 1) could then be an epiphenomenon explained probably by influence of the cancer cells on a residing viral genome, rather than the reverse conventional hypothesis that different E6 variants have a different potential to drive progression to invasive cervical cancer.

### ACKNOWLEDGEMENTS

Contract grant sponsor: Swedish Cancer Society grant No. 0055-B98-33XAB, contract grant sponsor: NIH grant No 1 RO:CA61197-01A3, contract grant sponsor: Russian Foundation for Basic Research grant 98-04-4993.

### REFERENCES

Alvarez-Salas L, Wilczynski S, Burger R, Monk B and Dipaolo J (1995) Polymorphism of the HPV-16 E6 gene of cervical carcinoma. *Int J Oncol* **7**: 261–266

Bontkes HJ, Van Duin M, de Gruijl TD, Duggan KM, Walboomers JM, Stukart MJ, Verheijen RH, Helmerhorst TJ, Meijer CJ, Scheper RJ, Stevens FR, Dyer PA, Sinnott P and Stern PL (1998) HPV 16 infection and progression of cervical intra-epithelial neoplasia: analysis of HLA polymorphism and HPV 16 E6 sequence variants. *Int J Cancer* **78**: 166–171

Bosch FX, Manos MM, Munoz N, Sherman M, Jansen AM, Peto J, Schiffman MH, Moreno V, Kurman R and Shah KV (1995) Prevalence of human papillomavirus in cervical cancer: a worldwide perspective. *J Natl Cancer Inst* **87**: 796–802

Dyson N, Howley PM, Munger K and Harlow E (1989) The human papilloma virus-16 E7 oncoprotein is able to bind to the retinoblastoma gene product. *Science* **243**: 934–937

Ellis JR, Keating PJ, Baird J, Hounsell EF, Renouf DV, Rowe M, Hopkins D, Duggan KM, Bartholomew JS, Young LS, et al. (1995) The association of an HPV16 oncogene variant with HLA-B7 has implications for vaccine design in cervical cancer. *Nat Med* **1**: 464–470

Eluf NJ, Booth M, Munoz N, Bosch FX, Meijer CJ and Walboomers JM (1994) Human papillomavirus and invasive cervical cancer in Brazil. *Br J Cancer* **69**: 114–119

Hedrum A, Ponten F, Ren Z, Lundeberg J, Ponten J and Uhlen M (1994) Sequence-based analysis of the human p53 gene based on microdissection of tumor biopsy samples. *Biotechniques* **17**: 118–119

Hu X, Guo Z, Tianyun P, Ponten F, Wilander E, Andersson S and Ponten J (1999) HPV typing and HPV16 E6-sequence variations in synchronous lesions of cervical squamous-cell carcinoma from Swedish patients. *Int J Cancer* **83**: 34–37

Londesborough P, Ho L, Terry G, Cuzick J, Wheeler C and Singer A (1996) Human papillomavirus genotype as a predictor of persistence and development of high-grade lesions in women with minor cervical abnormalities. *Int J Cancer* **69**: 364–368

Luxton J, Mant C, Greenwood B, Derias N, Nath R, Shepherd P and Cason J (2000) HPV16 E6 oncogene variants in women with cervical intraepithelial neoplasia. *J Med Virol* **60**: 337–341

Matsukura T and Sugase M (1995) Identification of genital human papillomaviruses in cervical biopsy specimens: segregation of specific virus types in specific clinicopathologic lesions. *Int J Cancer* **61**: 13–22

Mazurenko N, Attaleb M, Gritsko T, Semjonova L, Pavlova L, Sakharova O and Kisseljov F (1999) High resolution mapping of chromosome 6 deletions in cervical cancer. *Oncol Rep* **6**: 859–863

Munoz N, Bosch FX, de Sanjose S, Tafur L, Izarzugaza I, Gili M, Viladiu P, Navarro C, Martos C, Ascunce N, et al (1992) The causal link between human papillomavirus and invasive cervical cancer: a population-based case-control study in Colombia and Spain. *Int J Cancer* **52**: 743–749

Phelps WC, Munger K, Yee CL, Barnes JA and Howley PM (1992) Structure-function analysis of the human papillomavirus type 16 E7 oncoprotein. *J Virol* **66**: 2418–2427

Samoylova EV, Shaikhaiev GO, Petrov SV, Kisseljova NP and Kisseljov FL (1995) HPV infection in cervical-cancer cases in Russia. *Int J Cancer* **61**: 337–341

Sparen P, Gustafsson L, Friberg LG, Ponten J, Bergstrom R and Adami HO (1995) Improved control of invasive cervical cancer in Sweden over six decades by earlier clinical detection and better treatment. *J Clin Oncol* **13**: 715–725.

Stoppler MC, Ching K, Stoppler H, Clancy K, Schlegel R and Icenogle J (1996) Natural variants of the human papillomavirus type 16 E6 protein differ in their abilities to alter keratinocyte differentiation and to induce p53 degradation. *J Virol* **70**: 6987–6993

van Muyden RC, ter Harmsel BW, Smedts FM, Hermans J, Kuijpers JC, Raikhlin NT, Petrov S and Lebedev A, Ramaekers FC, Trimbos JB, Kleter B and Quint WG (1999) Detection and typing of human papillomavirus in cervical carcinomas in Russian women: a prognostic study. *Cancer* **85**: 2011–2016

von Knebel Doeberitz M, Rittmuller C, Aengeneyndt F, Jansen DP and Spitkovsky D (1994) Reversible repression of papillomavirus oncogene expression in cervical carcinoma cells: consequences for the phenotype and E6-p53 and E7-pRB interactions. *J Virol* **68**: 2811–2821

Werness BA, Levine AJ and Howley PM (1990) Association of human papillomavirus types 16 and 18 E6 proteins with p53. *Science* **248**: 76–79

Wheeler CM, Yamada T, Hildesheim A and Jenison SA (1997) Human papillomavirus type 16 sequence variants: identification by E6 and L1 lineage-specific hybridization. *J Clin Microbiol* **35**: 11–19

Williams C, Ponten F, Moberg C, Soderkvist P, Uhlen M, Ponten J, Sitbon G and Lundeberg J (1999) A high frequency of sequence alterations is due to formalin fixation of archival specimens. *Am J Pathol* **155**: 1467–1471

Xi LF, Demers GW, Koutsky LA, Kiviat NB, Kuypers J, Watts DH, Holmes KK and Galloway DA (1995) Analysis of human papillomavirus type 16 variants indicates establishment of persistent infection. *J Infect Dis* **172**: 747–755

Xi LF, Koutsky LA, Galloway DA, Kuypers J, Hughes JP, Wheeler CM, Holmes KK and Kiviat NB (1997) Genomic variation of human papillomavirus type 16 and risk for high grade cervical intraepithelial neoplasia [see comments]. *J Natl Cancer Inst* **89**: 796–802

Yamada T, Wheeler CM, Halpern AL, Stewart AC, Hildesheim A and Jenison SA (1995) Human papillomavirus type 16 variant lineages in United States populations characterized by nucleotide sequence analysis of the E6, L2, and L1 coding segments. *J Virol* **69**: 7743–7753

Yamada T, Manos MM, Peto J, Greer CE, Munoz N, Bosch FX and Wheeler CM (1997) Human papillomavirus type 16 sequence variation in cervical cancers: a worldwide perspective. *J Virol* **71**: 2463–2472

Zehbe I, Wilander E, Delius H and Tommasino M (1998) Human papillomavirus 16 E6 variants are more prevalent in invasive cervical carcinoma than the prototype. *Cancer Res* **58**: 829–833

© 2001 Cancer Research Campaign 
